# The association between body mass index and varicocele: A meta-analysis

**DOI:** 10.1590/S1677-5538.IBJU.2019.0210

**Published:** 2020-11-18

**Authors:** Guo Xiao-Bin, Wu Fang-Lei, Xia Hui, Yang Cheng, Cai Zhi-Xuan, Huang Zhi-Peng, Liu Cun-Dong, Guo Wen-Bin

**Affiliations:** 1 The third affiliated hospital of Southern Medical University Department of Urology Guangzhou China Department of Urology, The third affiliated hospital of Southern Medical University, Guangzhou, Guangdong 510630, P.R. China;; 2 The twelfth People's Hospital of Guangzhou Department of Stomatology Guangzhou China Department of Stomatology, The twelfth People's Hospital of Guangzhou, Guangzhou, Guangdong 528000, P.R. China

**Keywords:** Varicocele, Meta-Analysis [Publication Type], Body Mass Index

## Abstract

**Objective:**

Recently, several studies have found that obesity had a protective effect against varicocele, but no meta-analysis has confirmed this finding. Therefore, we conducted this meta-analysis to investigate the association between body mass index (BMI) and varicocele.

**Material and Methods:**

We searched for studies in PubMed, Science Direct and the Cochrane Library from inception until February 2018. The association between BMI and varicocele was assessed by pooling the odds ratios (ORs).

**Results:**

Eleven eligible studies with a total study population of 1.376.658 participants were included in our analysis. According to BMI, the subjects were defined as belonging to the obese, overweight and underweight groups. Our results showed that the obese group had a lower risk of varicocele when compared with the normal weight group (odds ratio [OR] 0.46, 95% confidence intervals [CIs] 0.37-0.58). Additionally, an overweight BMI had a protective effect against varicocele (OR 0.70, 95% CIs, 0.56-0.86). However, underweight patients had a more than 30% higher risk of varicocele (OR 1.31, 95% CI, 1.04-1.64). Furthermore, there was no publication bias in any of the analyses.

**Conclusions:**

Our study demonstrates that BMI is negatively associated with the presence of varicocele.

## INTRODUCTION

Varicocele is present in approximately 15% of the general population. However, more than one-third of men consult doctors regarding infertility, and nearly 80% of secondary infertile men suffer from varicocele (1). Varicocele is caused by dilatation and tortuosity of the pampiniform plexus. When the valves within the veins along the spermatic cord do not work appropriately, leading to blood backflow, varicocele occurs. The backflow of blood into the pampiniform plexus increases vein pressure and hypoxia, which may damage testicular spermatogenesis (2). It is well known that most patients have varicocele on the left side (3). Left renal vein entrapment, defined as compression of the left renal vein between the aorta and the superior mesenteric artery, is common in varicocele patients (4, 5). There were studies that showed that body mass index (BMI) was lower in patients with renal-vein entrapment than in controls, with a regression of haematuria correlating with an increase in BMI (6). Therefore, the relationship between BMI and varicocele is worth further discussion.

The prevalence of overweight and obesity has become a global problem. Overweight and obese are assessed by the body mass index (BMI), which is calculated as the weight (kg) divided by the square of the height (m^2^). It was expected that there would be more than 700 million obese adults and 2.3 billion overweight adults worldwide by 2015 (7). Recently, several studies (8-10) have found that obesity has a protective effect against varicocele, but no meta-analysis has confirmed this finding. Previous research has discussed the association between varicocele and other factors, such as height, age, lifestyle habits and BMI (11-16). There are inconsistent results regarding the relationship between varicocele and BMI. Some research suggested that BMI was inversely associated with the prevalence of varicocele (12-14), whereas other studies found no such relationship (10, 11, 17, 18). With this background, we conducted this meta-analysis to elucidate the relationship between BMI and varicocele.

## MATERIAL AND METHODS

### Search strategy

A comprehensive computerized search in PubMed, Science Direct and the Cochrane Library was conducted from inception to February 2018. We used the following search strategy: varicocele AND (body mass index OR BMI or underweight or obese or overweight). Reference lists and conference proceedings were also searched manually to identify possible additional studies.

### Study selection

The inclusion criteria were as follows: 1) the topic is varicocele; 2) odds ratios (ORs), relative risks (RRs), hazard ratios (HRs) and standardized incidence ratios with 95% confidence intervals (CIs) were provided or could be calculated; 3) randomized controlled trials or observational studies (case-control, cross-sectional or cohort studies) published as original studies to evaluate the association between BMI and varicocele; and 4) underweight, obese, overweight or BMI criteria were reported based on the definitions that were established by the Centers for Disease Control. Eligible studies were independently determined by two investigators (Guo Wenbin and Wu Fanglei). Differing decisions were resolved by mutual consensus.

Reviews, meeting abstracts, commentaries and editorials were excluded from our analysis. We also excluded the studies if they provided only an estimate of effect, with no means by which to calculate the standard error.

### Data extraction

A standardized data collection form was used to extract the following information: last name of the first author, year of publication, country of origin, study design, sample size, BMI category, and adjusted effect estimates with 95% CI. Two investigators (Yang Cheng and Huang Zhipeng) independently performed the data extraction.

#### Statistical Analysis

The strength of the relationship between BMI and varicocele was assessed by ORs. ORs were extracted from individual studies and were combined with a fixed-effect model or a random-effect model. Multivariate ORs were used for statistical analysis in preference to the univariate ORs. If the ORs were not directly provided, case and control group numbers were obtained. We first translated the data to ORs for further combination. The ORs from individual studies were transformed to their log [ORs] to stabilize the variance and normalize the distribution before pooling the studies (19). Pooled ORs <1 reflected a favourable outcome in obese patients compared with healthy subjects and indicated a lower morbidity rate.

For the meta-analysis, both the fixed-effects model (weighted with inverse variance) and the random-effects model were considered based on the level of heterogeneity. Pooled estimates of efficacy were calculated using the Mantel-Haenszel fixed-effects model first (20). However, if there was heterogeneity, the following methods were used to explore the source of heterogeneity: 1) a subgroup analysis and 2) a sensitivity analysis excluding the trials that potentially biased the results. If heterogeneity still existed, the DerSimonian and Laird random-effects model was used.

For each meta-analysis, we assessed the between-study heterogeneity using the X^2^ test and I^2^ statistics, which assessed the appropriateness of pooling the individual study results (21). The value of I^2^ indicates the degree of heterogeneity, with 0-25% indicating insignificant heterogeneity, 26-50% indicating low heterogeneity, 51-75% indicating moderate heterogeneity and more than 75% indicating high heterogeneity.

The presence of publication bias was assessed by funnel plots of the logarithm of the odds ratios versus their standard errors. We used Begg's (22) and Egger's (23) tests to evaluate the presence of publication bias in our primary end points; P <0.05 indicated bias, and P >0.05 indicated no publication bias. Stata 10.0 software was used for all the data analyses.

## RESULTS

**Figure 1:**
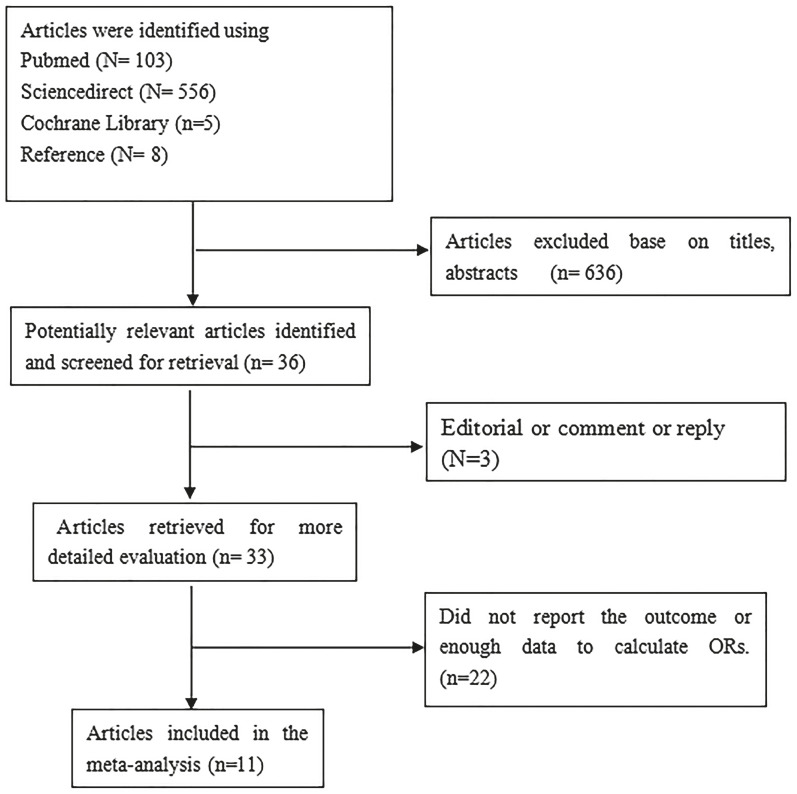
Flow chart for the selection of articles.

The search strategy generated 674 references: PubMed (N=103), ScienceDirect (N=556), and Cochrane Library (N=5). A total of thirty-six potentially eligible studies were identified by the literature search. Three articles were excluded because they were reviews, editorials and responses. We excluded twenty-two studies that did not report the outcome of varicocele or did not provide enough data to calculate the ORs. Finally, we identified eleven full-text articles (12, 14, 18, 24-31) that met the inclusion criteria. The search flow chart is shown in Figure-1, and the characteristics of the eleven included articles are summarized in Table-1. Of the eleven articles, eight were case-control studies (12, 14, 18, 25-29, 29, 30), and three (24, 28, 31) were cross-sectional studies. Three (12, 14, 25) were conducted in the US, four (26-29) in Europe, three (18, 24, 31) in Asia and one in Africa (30). The included studies were published between 2006 and 2017, with a total study population of 1.376.658 participants. The sample size of the studies varied from 98 (30) to 1.323.061 (28). The Newcastle-Ottawa scale was applied for assessment of quality of included studies in Table-3. As show, overall quality score of included studies were 8 or 9. This shows that the findings of these articles are trustworthy.

**Table 1:** Characteriste of the included studies.

Author / Published year	Country	Study design	Case	Control	BMI category
Nielsen ME et al. / 2006 (12)	USA	Case-control study	147	566	<25 Normal
			212	763	25-<30 Overweight
			34	295	30-<35 Obese
			5	82	>35 Very obese
Handel LN et al. / 2006 (13)	USA	Case-control study	378	506	<25 Normal
			540	1,009	25-<30 Overweight
			175	605	30–<35 Obese
Baek M et al. / 2011 (14)	South Korea	Cross-sectional study	205	783	<20 Underweight
			104	649	20-<25 Normal
			11	186	25-<30 Overweight
Chanc Walters R et al. / 2012 (15)	USA	Case-control study	129	245	<25 Normal
			163	372	25-<30 Overweight
			43	127	>30 Obese
Soylemez H et al. / 2012 (16)	Turkey	Case-control study	433	1,287	<25 Normal
			57	218	25-<30 Overweight
			8	58	>30 Obese
Gokce A et al. / 2013 (17)	Turkey	Case-control study	39	51	<20 Underweight
			290	527	20-<25 Normal
			208	509	25-<30 Overweight
			50	167	>30 Obese
Rais A et al. / 2013 (18)	Israel	Cross-sectional study	1,323	61	<5th percentile Underweight
					5th-84.9th percentile Normal
					85th-94.9th percentile Overweight
					≥95th percentile Obese
Do antekin et al. / 2014 (19)	Turkey	Case-control study	82	98	<25 Normal
			94	172	25-<30 Overweight
			34	120	>30 Obese
Loukil et al. / 2015 (20)	Tunisia	Case-control study	56	21	<25 Normal
			8	8	25-<30 Overweight
			3	2	>30 Obese
Shafi H et al. / 2015 (21)	Iran	Case-control study	153		<25 Normal
					25–<30 Overweight
					>30 Obese
Liu et al. / 2017 (22)	China	Cross-sectional study	39,559		<18.5 Underweight
					18.5–<25 Normal
					25–<30 Overweight
					>30 Obese

### Overweight and risk of varicocele

**Figure 2:**
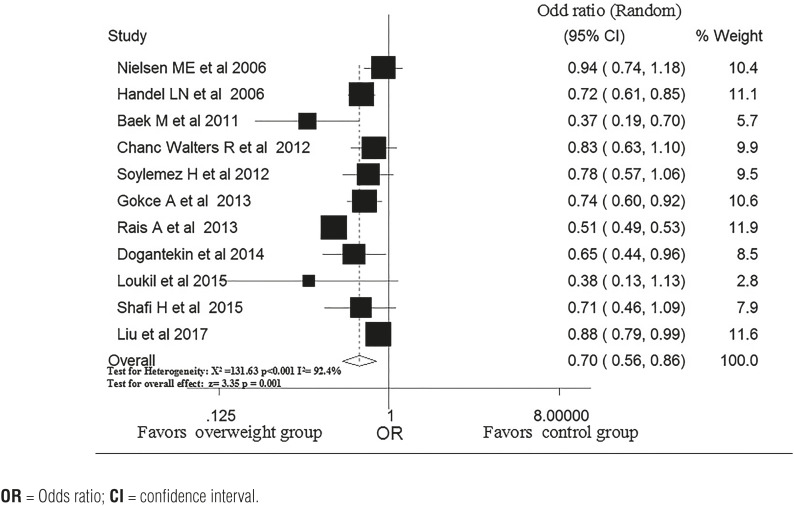
Pooled OR of varicocele in subjects with and without overweight.

The relationship between overweight and the risk of varicocele was explored in the eleven studies (12, 14, 18, 24-31). The ORs pooled by the random-effects model showed that overweight subjects had a lower overall risk of varicocele compared with healthy subjects (OR, 0.70; 95% CI, 0.56-0.86, P <0.001); Figure-2). There was significant heterogeneity in the pooled result (P for heterogeneity <0.001, I^2^=92.4%). In Rais's study, classification was carried out according to four groups: underweight (<5th percentile); normal weight (5th-84.9th percentile), overweight (85th-94.9th percentile) and obese (≥95th percentile), with normal weight as the reference group. An expanded analysis of the normal weight group included further classification into five percentile groups (5-9.9; 10-24.9; 25-49.9; 50-74.9 and 75-84.9), with 25-49.9 (the largest group) as the reference group. In the other studies, according to the National Institutes of Health definition, those patients with a BMI of less than 25kg/m^2^ were categorized as normal weight. Patients with a BMI of 25kg/m^2^ to less than 30kg/m^2^ were considered overweight, and those with a BMI greater than 30kg/m^2^ were categorized as obese.

### Obesity and risk of varicocele

**Figure 3:**
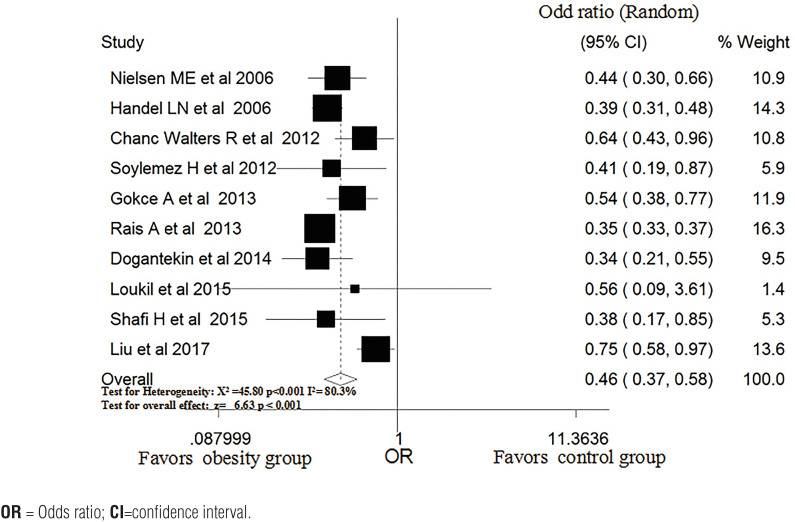
Pooled OR of varicocele in subjects with and without obesity.

Ten (12, 14, 18, 25-31) studies reported the relationship between obesity and the risk of varicocele. After pooling the data from these studies, the rate of varicocele was significantly lower in the obese group, and there was high heterogeneity among the studies (OR, 0.46; 95% CI, 0.37-0.58, P <0.001; P for heterogeneity=0.001, I^2^=80.3%; Figure-3).

### Underweight and risk of varicocele

**Figure 4:**
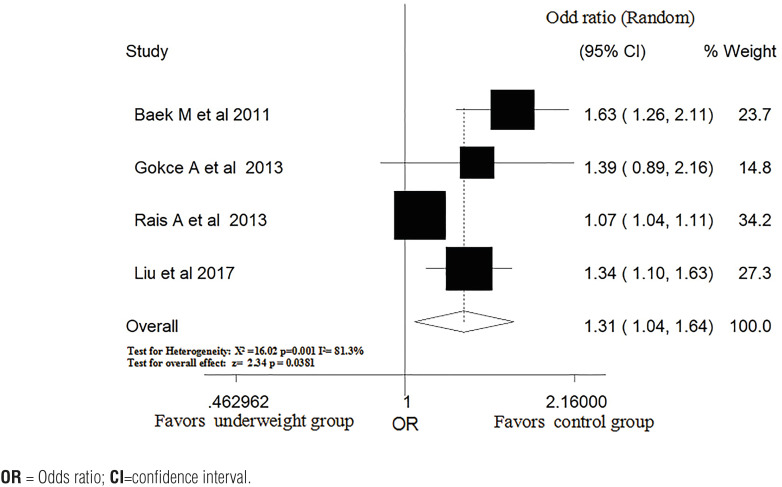
Pooled OR of varicocele in subjects with and without underweight.

Four (24, 27, 28, 31) studies reported the relationship between underweight and the risk of varicocele. The combined OR showed that the risk of varicocele was significantly higher in the underweight group than in the control group. There was high heterogeneity among the studies (OR, 1.31; 95% CI, 1.04-1.64, P=0.0381; P for heterogeneity=0.001, I^2^=81.3%; Figure-4).

A subgroup analysis was performed to investigate the source of heterogeneity in the overweight group according to study design and geographic location. Of the eleven studies, eight were case-control studies, and three used cross-sectional designs. The results derived from the subgroup analysis were consistent with the overall results, with ORs of 0.76 (95% CI, 0.69-0.84, P <0.001; P for heterogeneity=0.523, I^2^=0.0%) and 0.58 (95% CI, 0.37-0.93, P=0.024; P for heterogeneity=0.000, I^2^=97.5%), respectively, in the case-control and cross-sectional studies (Table-2).

**Table 2:** Summary of pooled ORs of BMI and risk of varicocele by subgroup analysis.

Subgroup		Number of studies	Pooled OR (95% CI)	Q-test for heterogeneity	
**BMI**				P value	I2 score
	Overweight (12-22)	11	0.70 (0.56-0.86)	0.000	92.4%
	Obese (12,13,15-22)	10	0.46 (0.37-0.58)	0.000	80.3%
	Underweight (14,17,18,22)	4	1.31 (1.04-1.64)	0.001	81.3%
**Study design (Overweight)**					
	Case-control study (12,13,15-17,19-21)	8	0.76 (0.69-0.84)	0.523	0.0%
	Cross-sectional study (14,18,22)	3	0.58 (0.37-0.93)	0.000	97.5%
**Geographic (Overweight)**					
	America (12,13,15)	3	0.79 (0.70-0.90)	0.185	40.7%
	Europe (16-19)	4	0.65 (0.50-0.85)	0.000	84.3%
	Asia (14,21,22)	3	0.67 (0.44-1.03)	0.024	73.3%
	Africa (20)	1	0.375 (0.125-1.128)	/	/
**Sensitivity analysis omitting Rais et al. study**					
	Overweight (12-17,19-22)	10	0.77 (0.69-0.86)	0.082	41.4%

**Table 3:** Newcastle-Ottawa scale for assessment of quality of included studies.

Quality assessment Criteria	Acceptable(*)	Nielsen ME 2006 (12)	Handel LN 2006 (13)	BaekM 2011 (14)		Chanc Walters R 2012 (15)		Soylemez H 2012 (16)			Gokce A 2013 (17)		Rais A 2013 (18)	Do anteki 2014 (19)			Loukil 2015 (20)		Shafi H 2015 (21)	Liu 2017 (22)
**Selection**												
**Representativeness of exposed cohort?**	Representative of average preemie in community (age/sex/being at risk of disease)	*	*	*		*		*			*		*	*			*		*	*
**Selection of the non-exposed cohort?**	Drawn from same community as exposed cohort	*	*	*		*		*			*		*	*			*		*	*
**Ascertainment of exposure?**	Secured records, Structured interview	*	*	*		*		*			*		*	*			*		*	*
**Demonstration that outcome of interest was not present at start of study?**		*	*	*		*		*			*		*	*			*		*	*
**Comparability**												
**Study controls for Age or sex?**		*	*	*		*		*			*		*	*		*		*		*
**Study controls for any additional factors?**		*	*	*		*		*			*		*	*		*		* **N/A**		*
**Outcome**												
**Assessment of outcome?**	Independent blind assessment, record linkage	*	*	*		*		*			*		*		*	*			*	*
**Was follow-up long enough for outcome to occur?**		*	*	*		*		*			*		*		*	*			*	*
**Adequacy of follow-up of cohorts?**	Complete FU, or subjects lost to FU unlikely to introduce bias	*	*	*		**N/A**		*			*		*		**N/A**	*			*	*
**Overall Quality Score (Maximum = 9)**		9 Good Quality	9 Good Quality		9 Good Quality		8 Good Quality		9 Good Quality	9 Good Quality		9 Good Quality			8 Good Quality	9 Good Quality		8 Good Quality		9 Good Quality

**FU** = Follow up; **N/A** = not applicable. Each asterisk represents if individual criterion within the subsection was fulfilled.

According to the geographic location analysis, the estimated ORs of varicocele in the overweight group compared with the normal group were 0.79 (95% CI, 0.70-0.90; P<0.001; P for heterogeneity=0.185; I^2^=40.7%) in the US, 0.65 (95% CI, 0.50-1.64; P=0.001; P for heterogeneity <0.001; I^2^=84.3%) in Europe and 0.67 (95% CI, 0.44-1.03; P=.070; P for heterogeneity=0.024; I^2^=73.3%) in Asia (Table-2). Begg's funnel plot and Egger's test were performed to assess the publication bias. The shape of the funnel plots did not reveal any evidence of asymmetry. The statistical results still did not show any publication bias (Begg's test P=0.062; Egger's test P=0.067).

## DISCUSSION

Obesity is associated with significant alterations in the hormonal milieu that can damage the reproductive system (32, 33). The relationship between obesity and fertility has received increased attention owing to the recent rapid increase in the prevalence of obesity worldwide, especially in developed countries (34, 35). Recent studies (13, 14, 24, 25) have found a lower prevalence of varicocele in obese patients. Consistent with most of these studies, our meta-analysis showed an inverse association between BMI and varicocele. With increasing BMI, the risk of varicocele decreases from 1.31 to 0.46 in individuals in the underweight and obese groups Our data showed that overweight people had a significantly lower incidence of varicocele, except for in the Asian population. Some previous studies in Asia found a similar phenomenon. In 2004, a study in Philadelphia (11) reported that patients with varicocele were significantly taller and heavier than those without varicocele, but there was no significant difference in BMI. In 2014, a Korean (10) study showed that the varicocele group had a significantly lower BMI in adolescents, but the difference was not significant in adults according to logistic regression analysis. Therefore, more studies are still needed to confirm the protective effect of obesity against varicocele in Asian populations.

Furthermore, the pooled ORs seem to show that the source of the heterogeneity was the study by Rais (28). When the study by Rais was omitted, the heterogeneity disappeared. The reason may be that the defined BMI categories in Rais's study were significantly different from those in other studies. In Rais's study, classification was carried out according to four groups: underweight (<5th percentile); normal weight (5th-84.9th percentile); overweight (85th-94.9th percentile) and obese (≥95th percentile), with normal weight as the reference group. An expanded analysis of the normal weight group included further classification into five percentile groups (5-9.9, 10-24.9, 25-49.9, 50-74.9 and 75-84.9), with 25-49.9 (the largest group) as the reference group. In the other studies, according to the National Institutes of Health definition, those patients with a BMI of less than 25kg/m^2^ were categorized as normal weight. Patients with a BMI of 25kg/m^2^ to less than 30kg/m^2^ were considered overweight, and those with a BMI greater than 30kg/m^2^ were categorized as obese.

Our meta-analysis showed an inverse association between BMI and varicocele. Two main theories have been postulated to clarify the inverse relationship between increasing BMI and decreasing occurrence of varicocele. One theory states that varicocele is caused by increased pressure in the left renal vein because it is compressed between the aorta and the superior mesenteric artery (36). Most researchers suggest that increased amounts of adipose tissue may decrease the compression of the left renal vein and provide a cushion, decreasing the nutcracker phenomenon in men with a higher BMI (12, 14, 37). Another theory believes that the detection of varicocele is decreased in men with a higher BMI because of the difficulty of palpation on physical examination due to the presence of adipose tissue in the inguinal and scrotal areas (14, 36). However, a recent study showed that obese patients had a lower prevalence of varicocele that was not due to difficulties with the physical examination caused by obesity. It is due to the decrease in the nutcracker phenomenon in men with a higher BMI (25).

This is a meta-analysis of observational studies with the limitations inherent in the study design. Therefore, at best, it can demonstrate an association but not a causal relationship. First, most studies calculated the ORs based on data without adjusting for confounding factors. Second, no prospective study could be included in the analysis, which may have biased the results. Third, some of the included studies had different BMI categories, which may confound the pooled results. Furthermore, these studies may have been vulnerable to surveillance bias, as patients with comorbidities would have been more likely to have follow-up imaging studies, leading to the more frequent detection of varicocele than in patients without comorbidities. Future studies that minimize these confounders and biases are needed to confirm this potential causal relationship.

Studies have shown that BMI could be a risk factor for left renal vein entrapment. In addition, our meta-analysis showed an inverse association between BMI and varicocele. Thus, for varicocele patients, especially those with lower BMI, attention should be paid to left renal vein entrapment.

It is well known that obesity is harmful to human health. The global obesity epidemic parallels a decrease in male fertility. However, the association between BMI and sperm parameters remains controversial. A study found that overweight and obesity are associated with an increased risk of azoospermia and oligozoospermia, which suggests that excess body weight affects sperm production (38). The inverse association between obesity and varicocele found in our study indicates that the causal relationship between obesity and poor sperm quality may be even stronger if the elevated risk of varicocele among lean males is taken into account. The diagnosis of varicocele in obese patients should be thoroughly discussed. Colour Doppler ultrasound (CDU) has the ability to detect the size of the pampiniformis plexus and blood flow parameters of the spermatic vein and is widely used in the diagnosis of varicocele (39). However, at present, there is a lack of completely standardized diagnostic criteria in obese men. We recommend using CDU to exclude nutcracker syndrome in patients with low BMI. It is of great value in the management of patients with different BMI varicocele, which can help find the cause of varicocele in some patients, so as to achieve better therapeutic result. Researchers have reported that ultrasound has a 95% sensitivity for the detection of a varicocele using a 2mm cut off for vein diameter (40). Pilatz reported that clinical varicocele can be predicted with high accuracy based only on the diameter of the testicular veins using cut-off values of 2.45mm at rest or 2.95mm during the Valsalva manoeuvre in the supine position (41). It would be more accurate in terms of diagnosing varicocele if patients were evaluated for reflux pattern, pampiniform venous plexus diameter, and venous reflux time (42). A study indicated that there was a significant correlation between the reflux pattern and two parameters of semen analysis, namely, sperm count and motility (42). Future research should explore the relationships between BMI and sperm parameters, male fertility and varicocele. Our study shows a significantly decreased risk of varicocele with increased BMI. However, this potential benefit should not be overemphasized, as obesity itself is harmful to the reproductive system. Is there any difference (s) in the workup and management of varicocele patients with different BMI? There is still not a clear answer. Future research should explore it.

## CONCLUSIONS

Our study shows a significantly decreased risk of varicocele with increased BMI. However, this potential benefit should not be overemphasized, as obesity itself is harmful to the reproductive system.
